# Determinants of retention in care of newborns diagnosed with sickle cell disease in Liberia: Results from a mixed-methods study of caregivers

**DOI:** 10.1371/journal.pgph.0001705

**Published:** 2023-04-04

**Authors:** Kanagasabai Udhayashankar, Patience D. Franklin, Cecelia J. Nuta, Adolphus K. Cherue, Heather Haq, Debbe Thompson, Venée N. Tubman

**Affiliations:** 1 Department of Pediatrics, John F. Kennedy Medical Center, Monrovia, Liberia; 2 Baylor College of Medicine International Pediatric AIDS Initiative, Texas Children’s Hospital, Houston, Texas, United States of America; 3 Department of Pediatrics, Baylor College of Medicine, Houston, Texas, United States of America; 4 USDA/ARS Children’s Nutrition Research Center, Baylor College of Medicine, Houston, Texas, United States of America; 5 Texas Children’s Cancer and Hematology Centers, Texas Children’s Hospital, Houston, Texas, United States of America; Yale University, UNITED STATES

## Abstract

High-income nations have established that early diagnosis and preventive treatment reduces early deaths in sickle cell disease (SCD). However, in low-/middle-income countries where SCD is common, attrition from clinical care is common. Reasons for poor retention in care are multi-factorial and poorly understood. The objective of this study was to identify factors that influence caregiver decision-making around chronic health care needs of a child with SCD. We conducted an exploratory sequential mixed methods study of caregivers of children diagnosed with SCD during a newborn screening program in Liberia. Caregivers completed questionnaires and semi-structured interviews designed to identify drivers of health decision-making. Interviews were digitally recorded, transcribed, coded, and analyzed using semi-structured thematic analysis to identify themes. Data integration occurred by using quantitative results to expand and clarify the qualitative themes. Twenty-six caregivers participated in the study. The mean age of the child at the interview was 43.7 months. Five themes influencing health decisions were identified: grief, the importance of support networks, stigma, perceived benefits, and the burden of chronic disease. The five themes crossed multiple domains of a socioecological model and identified complex interactions between family, community, social and cultural norms, and organizational structures. This study highlights the importance of community awareness of SCD and appropriate health communication by healthcare workers. Healthcare decision-making is multifactorial and complex. These results provide a framework for improving retention in care. In a low-resource country such as Liberia, much can be done by leveraging existing resources and cultural practices.

## Introduction

Sickle cell disease (SCD) is an inherited disorder of hemoglobin. In the WHO Afro Region, 10 to 40% of the population in some countries carry a sickle cell gene resulting in an estimated disease prevalence of 1–2% [1, 2]. In 2006, the World Health Organization (WHO) described SCD as a public health priority for the region as SCD contributes to about 9–16% of under-5 mortality in West Africa [2]. Over 300,000 infants are born with SCD in sub-Saharan Africa (SSA) annually. While survival to age 5 years is estimated to be 95% for children in high-income countries, only 10–50% of those in SSA survive to 5 years of age [2–4]. In 2010, WHO drafted a public health strategy to reduce the burden of SCD in SSA through improved awareness, disease prevention and early detection [1]. Recommended interventions included improvements in health-care provision; clinical, laboratory, diagnostic and imaging facilities adapted to different levels of the health system; screening of newborns; training of health workers; genetic counselling and testing; accessibility to healthcare; establishment of patient support groups; advocacy; and research. Those life-saving interventions have been difficult to implement.

Understanding that early detection improves survival, a pilot newborn screening program for SCD was implemented in Liberia in 2012. The 2012 pilot study found sickle cell trait occurred among 10.3% of study participants and SCD occurred in 1.2% of the study population [5]. Liberia is a low-income country in West Africa with a population of approximately 5 million. Approximately 54% of the population lives below the poverty line and 83.7% of the population lives on less than $2 a day [6, 7]. By 2003, 14 years of civil war had left Liberia with a crumbling infrastructure and one of the weakest health systems in the world. In 2014, the Ebola Virus Disease outbreak further battered the health system [8, 9] and forced the pilot program to pause. During the pilot program, all newborns diagnosed with SCD through newborn screening were referred to the specialty SCD clinic at the national referral hospital. All patients referred received education, counseling, and penicillin prophylaxis. The pilot program has since been restarted and expanded to other sites in the country [10].

Without ongoing preventive care, it is unclear whether the survival gains from newborn screening would persist. Within four months of diagnosis in the pilot program, 79% of families had returned to the SCD clinic for at least one visit. Unfortunately, by six months after diagnosis, 75% of families were lost from regular care. The aim of this study is to identify factors that influence caregiver decision-making around the chronic health care needs of a child diagnosed with SCD through the NBS and referred for follow-up care.

## Methods

### Study participants and recruitment

The study design was exploratory sequential mixed methods. A convenience sample of caregivers of children diagnosed with SCD through the pilot program at John F. Kennedy Medical Center (JFKMC), the teaching and referral hospital in Monrovia, Liberia was recruited. Caregivers who either regularly attended the SCD Clinic or those who were considered lost to follow-up and had either attended once or never attended were also included in the study. A caregiver was invited to participate in the study through contact by telephone, text message, in person at clinic visits, or during a home visit. A caregiver was eligible to participate if he/she self-identified as the primary caregiver, were aged 18 years or above, and had sufficient English language comprehension to participate in the interview and complete the questionnaire. There were no specific exclusion criteria. One caregiver per patient was recruited. Caregivers received remuneration for their time and travel.

### Data collection

The research question guiding this investigation was whether factors that drove decision-making for families with children with SCD could be modified to improve retention in care and ultimately, to improve patient outcomes. A semi-structured interview guide was designed by the authors to elicit knowledge, perceptions and behaviors of caregivers related to their child’s SCD diagnosis and care. The guide was designed to assess five domains (intrapersonal, interpersonal, community, external, and organizational) of decision-making as part of a socio-ecologic model outlined in the pediatric HIV literature [11]. The socio-ecologic model takes into consideration the complex interplay of factors between individuals, families, communities, and society. Interviewers were healthcare workers trained in qualitative interview techniques. Interviews were conducted in private, between November 2016—April 2017. All interviews were conducted in English. Interviews were digitally recorded and transcribed verbatim. Examples of interview questions were: “Please describe your experience receiving the diagnosis of SCD?” and “What are some of the barriers preventing you from attending the clinic?”.

Prior to the interview, demographic information about the participant and patient were solicited with a 56-item questionnaire, along with information about family dynamics. The questionnaire was read aloud to participants. Visits to the JFKMC were validated by medical record review. Dates and ages were verified by comparing to records obtained in the newborn screening program [5].

### Ethics

All study staff completed human subjects research training prior to the initiation of the study. Due to low literacy levels and concerns about comprehension the consent was read aloud prior to all participants providing consent. Written informed consent was obtained from all participants. The research was conducted in accordance with ethical research practices and reviewed by the Institutional Review Boards at University of Liberia—PIRE and Baylor College of Medicine.

### Data analysis

Prior to analysis of qualitative data, transcripts were cross-checked for accuracy by one author who was also an interviewer (VNT). Final transcripts were uploaded into Dedoose Version 8.035. An initial codebook containing codes and corresponding definitions was developed by adapting a socioecological framework developed for HIV research for SCD [11]. Within each of five domains are subdomains that more specifically characterize drivers of decision-making.

Using the initial codebook as a guide, transcripts were coded by two independent coders (KU, HH) trained in qualitative research methodology using a hybrid thematic analytic approach [12]. This approach includes both structured and emergent codes. Structured codes were derived from the adapted socioecological model [11], supported by emergent codes which were generated during coding to capture additional features of the data in relation to the research question. Once finalized, each coder then used the codebook to independently apply codes to the transcripts, meeting periodically to compare. A third author (VNT) served as a tie-breaker for any disagreements. After coding was complete, codes were reviewed to identify, define, refine, and name themes and subthemes.

For quantitative data analysis, the International Wealth Index (IWI) calculator was used to determine the average household wealth of the study participants based on questionnaire responses [13]. The IWI is scored from 0–100, with 0 representing households with none of the assets in question and lowest quality housing and 100 representing households having all assets and highest quality housing. These assets include seven consumer durables (possession of a TV, fridge, phone, bike, car, a cheap utensil and an expensive utensil), access to two public services (water and electricity) and three housing characteristics (number of sleeping rooms, quality of floor material and of toilet facility). In 2020, the average national IWI for Liberia was 32.6, with a range across counties of 18.8 in Gbarpolu to 45.7 in Monteserado. Data from questionnaires were entered into RedCap [14] and descriptive statistics summarized using Microsoft Excel or GraphPad Prism. Data integration occurred by using the quantitative results to confirm and expand qualitative findings.

## Results

**Fig 1:**
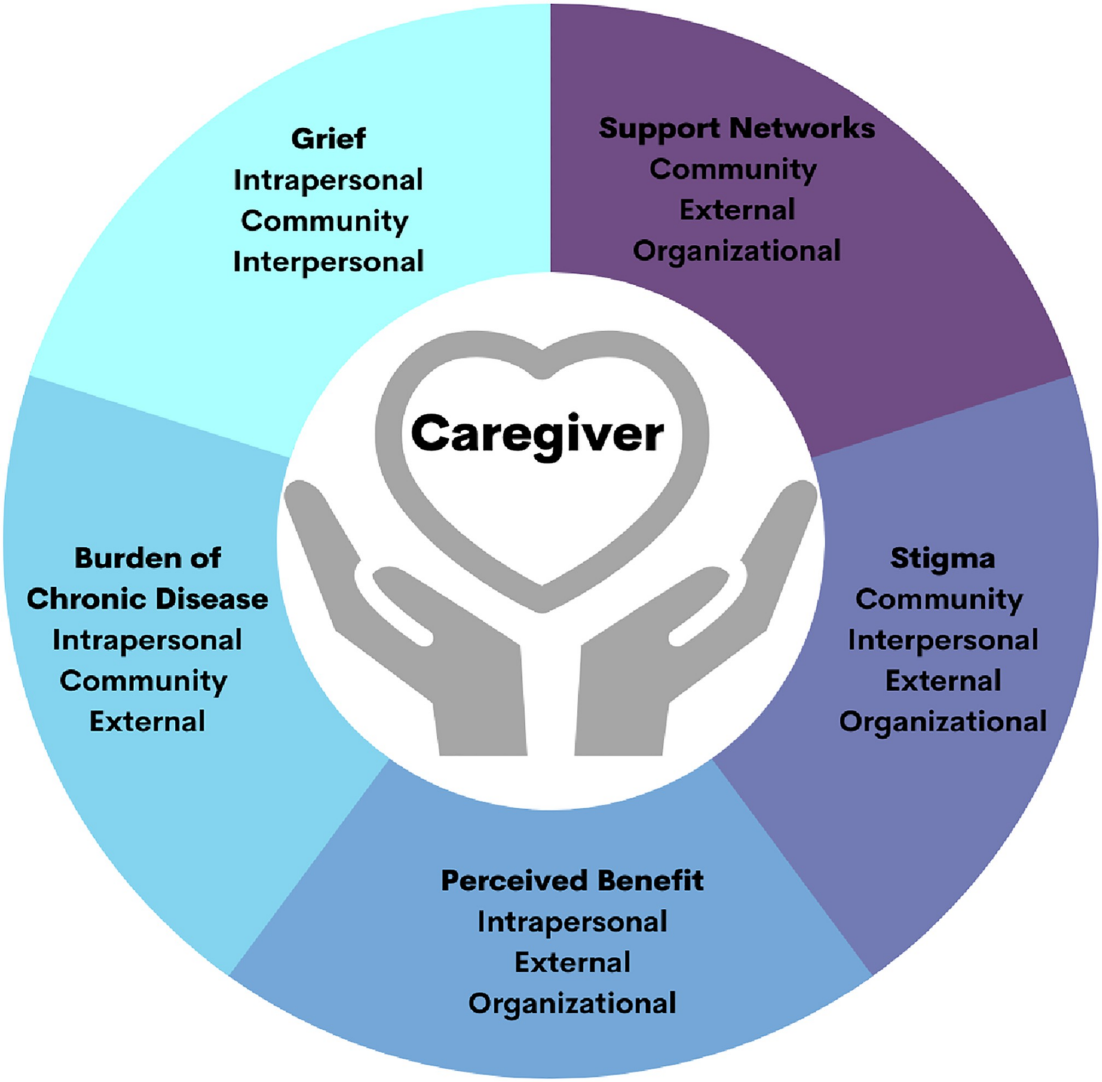
Conceptual model of drivers of healthcare decisions for caregivers of children with sickle cell disease.

From the primary study, 28 of 38 caregivers whose infant received a SCD diagnosis were approached and 26 (92.9%) were recruited (S1 Fig). The median age of caregivers was 33.5 years (IQR: 9.5 years), with 85% of participants being female (Table 1). The median age of the child at the time of interview was 46.5 months (IQR: 7.25 months), with 48.1% being female. Most caregivers were biological parents (77.8%); however, others were grandparents, aunts, or guardians. Most participants had completed high school (46.2%) or primary school (26.9%), with a minority having completed college or higher (11.15%). After coding and analysis were complete, five themes emerged: grief, stigma, burden of chronic disease, support networks, and perceived benefits. The themes were arranged into a conceptual model (Fig 1). Within each theme, we identified common elements of the socioecological model reported by the caregivers. Themes and cross-cutting common elements are represented by verbatim quotes (Table 2).

**Table 1:** Characteristics of caregivers and SCD patients.

Variable	% (n)
**Interviewer Characteristics**	
Age (mean)	33.3 years
Sex	
Female	84.6 (4)
Relationship to child	
Biological parent	76.9 (20)
Guardian	3.8 (1)
Other Family Member	19.2 (5)
Living with child	
Yes	88.5 (23)
Employment status	
Student	26.9 (7)
Working part-time or full-time	23.1 (6)
Not working	50. (13)
Highest level of education completed?	
Informal or primary	26.9 (7)
High School / Secondary	46.2 (12)
Vocational school/ some college	15.4 (4)
College degree or higher	11.5 (3)
Religious affiliation?	
Christian	92.3 (24)
Muslim	7.7 (2)
Marital status	
Married	34.6 (9)
Never married	61.5 (16)
Widowed	3.8 (1)
**Patient Characteristics**	
Child’s age (mean)	44 months
Child’s sex	
Female	46.2 (12)
What is your child’s diagnosis?	
Sickle cell disease, not specified	84.6 (22)
Hemoglobin SC	3.8 (1)
I don’t know	11.5 (3)
**Access to Care**	
Mode of transportation to clinic	
Walk	11.5 (3)
Public transportation	73.1 (19)
Private transportation	15.4 (4)
Time from home to clinic	
Less than 30 minutes	23.1 (6)
31–60 minutes	19.2 (5)
Over 60 minutes	57.7 (15)
**Health System Interactions**	
Number of visits since diagnosis	
Never returned	23.1 (6)
0–5 visits	42.3 (11)
6–10 visits	15.4 (4)
>10 visits	19.2 (5)
Does your child take penicillin?	
Yes	53.8 (14)
Vaccination status	
Fully vaccinated	92.3 (24)
Partially vaccinated	7.7 (2)
How often do you treat your child for malaria	
When they have a fever	53.8 (14)
Every 3 months (as recommended)	26.9 (7)
Other	19.2 (5)

**Table 2:** Representative quotes demonstrating emergent themes and corresponding socioecological levels. Quotes are identified by parent sex and child age at time of interview, and are arranged according to the socioecological model (Table 2).

Theme	Socio-ecological level	Representative Quotation
**Grief**	***Community***	“I started giving up on my daughter after all I heard from [the doctor].” [mother of 45-month-old child]
	***Interpersonal***	“When I went to her father I called him … I asked him, I went there in anger, because I was angry when I went there. I asked if he has sickle cell, he said no. In fact, [he said] he passed 21 years [so] he can’t get sickle cell [anymore].” [mother of 45-month-old child]
	***Intrapersonal***	“I don’t believe [the diagnosis]. I don’t want to pronounce it on my child.” [mother of 53-month-old child]
**Stigma**	***Community***	“If you I start explaining it to them …they will carry it and to explain to … another person.” [mother of 45-month-old child]
		“They will always make you to feel discouraged by telling you…, ‘That sickness, that not good. It can’t make the child to stay long in your hand and the child will die soon so, I felt it was not good to tell anyone or share with people.” [mother of 46-month-old child]
		“No, not really people in the community because sometimes, when people have sicknesses like this, it’s like… I don’t know how today it… but it’s like, sometimes the people will be like a stigma. Some people, when you’re walking, they will try to mock you. So, only people you can trust are those people you can confide in.” [mother of 48-month-old child]
	***External***	“My heart cut… taking sickle cell and AIDS to be the same… I think sickle cell can kill so soon. So, I thought she [would] get some kind of sickness that within a second, she will just vanish. [mother of 46-month-old child]
	***Organizational***	“Sometimes I communicated it to my niece. She is working with Catholic [Hospital]. She gave me a shock; she says ‘… Say how? That thing there, it’s hard to [cure] people! It’s got to kill you anyhow!’. I say, ‘What! How you will talk like that?’. She say, ‘As far as I’m concerned, that is it.’ So, I usually don’t share it with people before they discourage me.’ [father of 42-month-old child]
		‘It didn’t really get to my mind to say … when I go to the other hospital, I should tell them.” [mother of 48-month-old child]
**Support networks**	***Community***	“Because I never wanted people to be looking at my son like he’s not… he don’t have a long time on planet Earth and [quiet]. I only shared it with my family and my pastor.” [mother of 51-month-old child]
		“But I started asking my wife, you know, I started asking, then I myself, I started checking my family record. … there was no information like that. Then the next thing that came to my mind is, I say, could it be that these people [hospital lab] mismatched the boy sample with another person?” [father of 36-month-old child]
		“The whole sickness [is] the thing that’s giving me a hard time… the finances to carry him to hospital because I [don’t have] money to carry him to the hospital. So, I carry him to the church, then they take him to a lady in the church. She’s a doctor. So, she’s the one… when I [don’t have it] at all, when she’s not around, then [*voice trails off*]…” [mother of 48-month-old child]
	***External***	“For me, from my wife and myself, I discuss [my son’s care] with my older brother, my older sister, and second, tell my pastor.” [father of 50-month-old child]
		“I actually talked to my pastor concerning it… They say, when one is sick, they should go to the elders and let the elders pray for them. I think with the prayers of the pastor, God’s intervention, I believe that things will be ok.” [mother of 48-month-old child]
		“For me, I don’t really see anything hard [about caring for him]. Because, you know, he’s my child and coming down with that disease… the only thing I have to do is to pray to God and continue to give him his medication.” [mother of 48-month-old child]
		“The process, like, I’m saying mean okay… like, when the appointment time is today. When I come, y’all will I ready to see doctors. Doctors will be able to cater to her, to do her checkup and give her medications.” [mother of 45-month-old child]
		The only experience that I have was a doctor/nurse here that used to call me… Even if I [was] forgetting, she [was] always reminding me that I should I bring my son for treatment. She always used to call me. … she said she no longer work here… So, since then, I never have brought my son here for checkup [mother of 53-month-old child]
	***Interpersonal***	“This kind of sickness it’s not good to keep it to yourself. It’s good to tell the father about it or if the father is not around and you have a sister of friend, tell them about the sickness.” [mother of 48-month-old child]
		“[The child’s] father and my mother [help make decisions about her health]. Because My mother is taking care [and] her father facilitates and helps with financing.” [mother of 50-month-old child]
**Perceived benefit**	***External***	“[Coming to sickle cell clinic] will help your child. Because once the child is sick and you bring the child to the hospital, the treatments, the medication they will give the child [will] help the child to recover quickly.” [mother of 52-month-old child]
		“Carrying the child to the clinic, the doctor will… when the child come down with the sickle cell, the doctor will diagnose… maybe the medicine will help the child out." [mother of 46-month-old child]
		“It’s important to bring him because I think they can prescribe a treatment for his sickness." [mother of 51-month-old child]
		“[I’m not a] doctor to know what [is] happening to my child. Since y’all said my child got sickle cell, I will be bringing him. So every time when the time reaching for his… for his… the treatment, they can call me and I can bring him.” [mother of 43-month-old child]
	***Intrapersonal***	"Yes. It was somewhat difficult for [my family] to accept [the diagnosis] but, they said I should always bring him here. Anytime. And my sister is really in support of me, always bringing him here. Anytime he even feels… falls sick, I shouldn’t buy him any medicine from drugstores, I should always bring him. " [mother of 51-month-old child]
		“Besides that [child will] live for long? Yeah. The benefit is to come for treatment." [mother of 52-month-old child]
		“Because I feel if I bring him, I will get better treatment for him to live longer.” [mother of 53-month-old child]
	***Organizational***	“Anytime I bring my child to the clinic I can’t find it difficult…. It can be very easy. I can see the doctor. They can ask me, I explain what happening, they can check him, they give his treatment. I find it very easy.”[mother of 45-month-old child]
		[When the doctor said my child has sickle cell] I… I… I felt bad because I tell doctor ‘That one too big for me.’ …I rebuke it. [mother of 50-month-old child]
		[The first time I was told that my daughter has sickle cell], “it was difficult because [exhales], …because as I learned, those with sickle cell … can’t pass certain age. I think they say 25? They can’t live longer, really.” [mother of 48-month-old child]
		“[The clinic nurse] used to call me always and I would bring him for his treatment. So, I ask all parents: if they diagnose your child or children, please come for the treatment. Whether the child had the sickness or they don’t have it, just come. You should come, that’s only God can help to save the child.” [mother of 43-month-old child]
		"When it started first, it was looking like a challenge for me. But whenever long we come, things going easier for us. When we come like that, so long we bring our chart [garbled] when they know are chronic care patient, there is no hauling and pulling. We can come directly and they can see us and give us our treatment and we go back." [mother of 45-month-old child]
		“If I bring my child here, they take good care of my child. The nurses, the doctors that [are] on duty, they take care of my child.”[mother of 52-month-old child]
**Burden of chronic disease**	***Community***	[My advice to other parents] They will have to just be calm… Especially the mother: you have continue to counsel [the mother] because sometimes when the child goes into crisis, you know, it’s like you are so disturbed. You are so disturbed. But with the encouragement, at least it can help build you up. [mother of 48-month-old child]
		“I told my sister, it was not hard for me, When I told her, she shout. ‘Ahh! That kind of sickness! You really need to be prayerful.’ That’s what she said. I said all day, God is in charge. But nothing will happen to him. I told her.” [mother of 48-month-old child]
	***External***	… sometimes the financial aspect [stops us from coming to clinic]. When you do not [have money], you will not be able to come to the clinic. Because when you come to the clinic, [it costs] money. That’s why sometimes, we can’t come. [grandmother of 47-month-old child]
		Sometimes finances, transport … prevent me [from coming to clinic]. [mother of 53-month-old child]
		Because to get [public transportation] to get here is not easy. Even if God bless when you get a [taxi], the traffic will continue to block until [the child’s mother] will get here late. …And also to go back home, to get [a taxi]: one time she almost slept here [at the hospital]. Thank God: … somewhere around 8, 9 [pm], she got something and she got home. [father of 42-month-old child]
	***Intrapersonal***	“…sometime if we don’t get [money] or car, truck, transportation that will really make them not to come. It’s is one of the challenges that we are facing because … as I said before I’m not working. And my wife and the other children are all on me. So those are the challenges that we can face. [father of 42-month-old child]
		Aah… it can be difficult because the transportation, mainly standing for car. Sometime the traffic and when you come, the line can be very long. [grandmother of 47-month-old child]
		The finances to carry him to hospital because I can’t be having money to carry him to the hospital so I carry him to the church then they take him to a lady in the church, she’s a doctor. So she’s the one… when I [don’t have it] at all, when she’s not around, then … [mother of 48-month-old child]

### “I Do Not Believe It”: The stages of grieving inform caregivers’ health-seeking behavior

The emotions experienced by many caregivers between diagnosis and the interview aligned with stages of grieving and influenced retention in care. Grief manifested through intrapersonal, community factors, and interpersonal factors (Fig 1). Although there was wide variation among the caregivers in the description of the manifestations of grief, common threads surfaced among the interviews representing the stages of grief, including denial, anger, bargaining, depression, and acceptance.

Most caregivers demonstrated disbelief and shock upon disclosure of the SCD diagnosis (Table 2). In the questionnaire, 16 of 26 participants reported knowing another person with SCD, including 6 with an affected family member and 2 with a death in the family attributable to SCD (Table 1). Many refused to believe the diagnosis and reported that SCD was unfamiliar to them. Caregivers reported that their newborn had appeared healthy and thus, the diagnosis was inconsistent with that appearance. They reported that their extended family members were healthy, and thus they perceived their family to not be at risk for SCD. Caregivers expressed anger at family members and spouses who did not disclose that they were carriers of sickle cell trait, those who did not know their sickle cell carrier status, and those that had knowledge that the disease existed in the family. Caregivers described feelings of helplessness, often voiced in conjunction with fear of having a chronically ill child. Underlying the experiences relayed in the interviews were feelings of guilt for many caregivers. Most caregivers evolved toward acceptance of the diagnosis, generally in association with highlighting their role the importance of seeking care and medications. Where family support was available, the caregiver was able to more quickly move toward acceptance.

Several community-oriented factors highlighted grieving as both internally driven and externally amplified or mollified. Interwoven into the theme of grief, caregivers expressed concerns for how disease disclosure occurred or was delivered by a provider, as well as factors such as misinformation and myths about SCD that are prevalent in the community. Strong cultural and social links to religion enabled consultation with pastors and faith leaders to be a mechanism for accepting the diagnosis of chronic disease. All participants identified a religious affiliation (Table 1). Caregivers shared how faith communities provided a source of hope and space for non-judgmental disclosure of disease.

### “The Child Will Die Soon”: Misconceptions drive stigma towards children, parents and families

While most study participants did not use the word stigma, they recounted negative experiences with community, interpersonal and structural/organizational factors which impacted healthcare decisions (Table 2). External factors such as the caregiver’s gender contributed to stigma. Many of the interviewed caregivers assigned blame to the mother for the diagnosis, misunderstanding the autosomal recessive nature of the disease. This was true for caregivers who were and those who were not the mother of the child.

Caregivers internalized stigma, which heightened social isolation, anxiety and despair. While 84.6% of participants reported disclosure to family, only 46.2% reported disclosing the diagnosis to friends (Table 1). Caregivers alluded to the similarity between SCD and other diseases associated with stigma and discrimination, such as HIV. A prevalent and damaging example is the myth of death around 18 years of age. This myth in particular led to feelings of shame and made caregivers reticent to disclose the child’s diagnosis for fear of stigmatization of themselves, the child, and their families. Caregivers suggested that their lack of knowledge and understanding of the disease made it difficult to disclose and discuss the diagnosis with family and community.

Some caregivers described receiving misinformation not just from the community but also from within the health system, including from within JFKMC and from other facilities. Perpetuation of misinformation within the health system itself served as a source of stigmatization and a deterrent to accessing the health system. Despite caregivers acknowledging the importance of seeking care and the severity of the disease, they reported being told not to accept the diagnosis for their child. Due to the fear of stigma by outsiders, a large number of caregivers saw continuity of care with known health providers to be safer and reduced fears of being stigmatized by the health system. This behavior led to non-disclosure of child’s SCD status to providers at other health facilities, increasing the likelihood for mismanagement of the condition.

### “I Don’t Have Money”: The burdens of caring for a child with SCD affects access to healthcare

Caregivers described complicated financial, psychological and emotional burdens of caring for a child with a chronic disease which altered decisions around retention in care. The participants included 26.9% students, 23.1% working at least part-time, and 50% not working. Despite a recommended quarterly visit schedule, 23.1% of participants never returned to the SCD clinic after the initial visit, 42.3% had fewer than 5 visits over 4 years, and 19.2% had more than 10 visits in the observation period. This differs from frequent interactions with the healthcare system for routine childhood immunizations (Table 1). The burdens of the diagnosis manifest through intrapersonal, community, and external factors.

**Fig 2:**
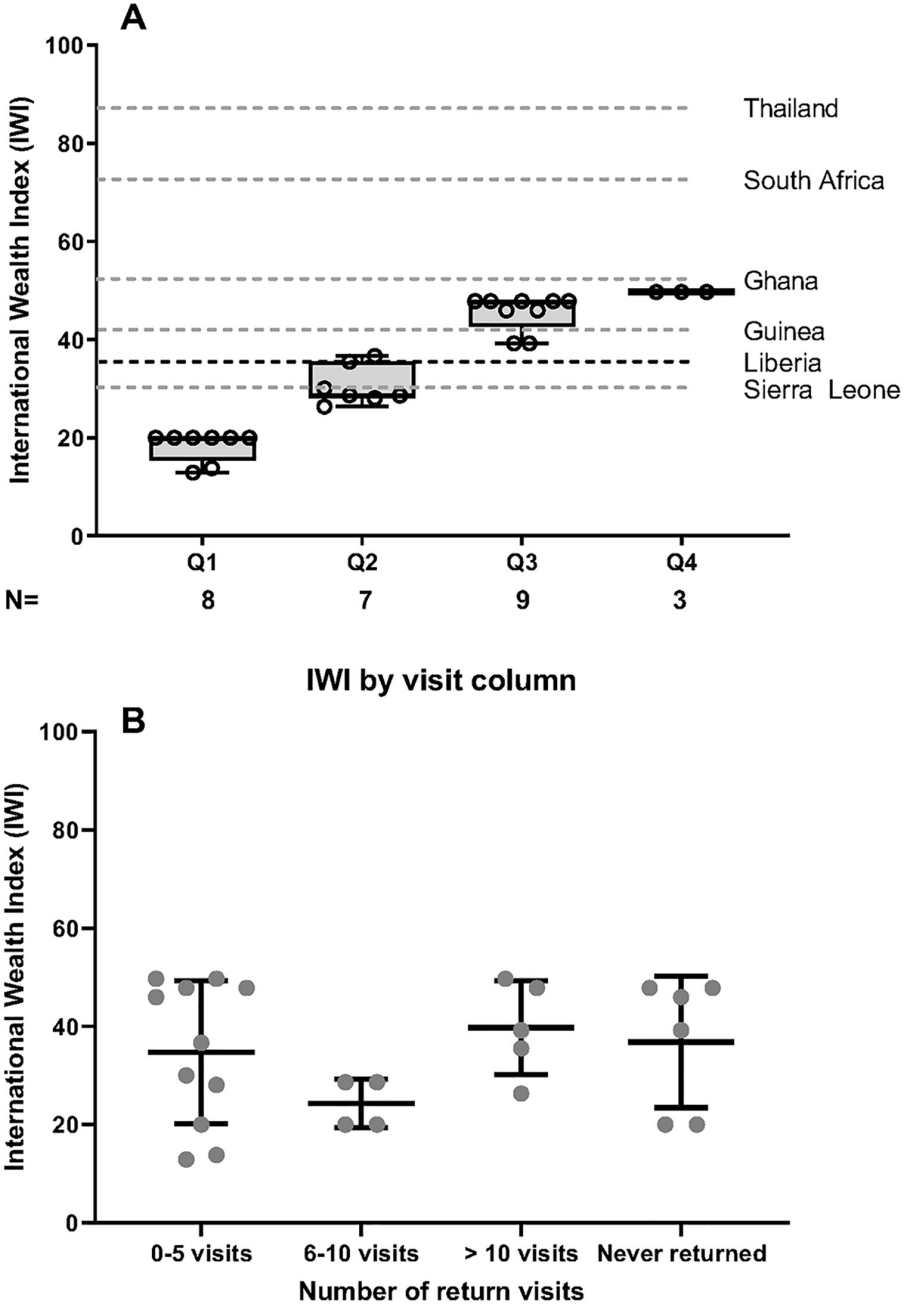
International Wealth Index by quartile and return visit. **A:** Box and whisker plots representing the distributions of IWI scores for study participants. Dashed lines represent the mean IWI score for all of Liberia, along with means for selected regional, continental, and international low- and middle-income countries. **B:** Scattered plot depicting IWI scores for participants against number of return visits. Mean with standard deviation shown. Dashed line represent the mean IWI score for all of Liberia.

The economic status of the family contributed to healthcare decisions. The median IWI for study participants was 35.5 (Fig 2A). Monetary costs associated with the need to frequently access healthcare, such as costs of medications or visits and cost of transportation determined whether or not they were able to make a clinic appointment. There was no association between IWI and the number of return visits (Fig 2B). Of 26 participants, 73.1% relied on public transportation (Table 1). The logistical costs of accessing care included distance from place of residence to that of the health facility, the time spent in transit, and time spent away from work. For transportation to the hospital, 57.7% reported spending greater than 60 minutes in transit to the hospital for an appointment (Table 1).

Caregivers expressed a feeling of being overwhelmed by the diagnosis, that the disease was “too big” or too serious. Caregivers reported being told not to “pronounce” such a diagnosis of their child. Sometimes, this advice came from community members, and sometimes it appeared as a representation of denial. Caregivers feared the voicing the diagnosis would lead to true manifestation of the disease. The psychological burden of the diagnosis was made greater by the perception that they were giving the diagnosis, rather than the diagnosis already existing. This emotional burden led to some avoidant behaviors. Some caregivers endorsed not wanting to be seen going to the hospital.

### “Through God’s Will, the Child Will Survive”: Religious beliefs and cultural support networks serve as positive and negative drivers

Support networks for caregivers emerged as a key factor in decision-making. Systems within communities, religious organizations, and health healthcare organizations influenced health seeking behavior positively, by sustaining caregivers emotionally and financially, and influenced negatively, by perpetuating myths about SCD (Table 2).

Family and community networks negatively impacted caregiver decision making by perpetuating prevalent community misconceptions about SCD. Fear of stigma and discrimination led to failure to seek care or failure to disclose the diagnosis at outside facilities. Denial of the child’s diagnosis led to delays in care. Additionally, misinformation related to the survival of children with SCD enhanced a perception of futility of medical intervention.

Caregiver decisions were positively influenced by support networks to manage the diagnosis of SCD. The interviews captured how community and family support helped to reduce feelings of shock, grief, disappointment, and despair (Table 2). Strong religious beliefs and faith communities provided a supportive environment that both individuals and families relied on as they dealt with the chronic disease diagnosis. Caregivers were encouraged to seek care for their child and to continue to care for their child despite the diagnosis. Despite expressing fears of disclosing the disease to community members, most caregivers acknowledged the importance of confiding in faith leaders.

Caregivers revealed how family members and faith communities were critical in providing financial support to access healthcare. This includes gifts of transportation and payment for hospital services and medications. The enormous costs of caring for a chronically ill child in an LMIC were apparent in all interviews. Obvious costs such as transport, hospital fees, and medications were addressed. However, the costs of care for other children, missed wages, and time were also frequently mentioned.

Caregivers also highlighted organizational support such as personal connection with the healthcare team influencing their decision-making processes and contributing to continuity of care. Even years after an encounter, caregivers could recount an experience with a specific healthcare provider who gave knowledge or encouragement for an intervention. Caregivers perceived clinic attendance to be beneficial for medical assessment and advice. Intertwined with this was the perception that medications provided by the health system was beneficial.

### “I will get better treatment for him to live longer” The perceived benefits of treatment versus the role of medications

Perceived benefits of healthcare emerged as a theme related to healthcare decision-making at intrapersonal, external, and organizational levels. Most caregivers saw the benefit of accessing free or subsidized medications and access to a physician who understood SCD. Caregivers perceived that the clinic visits, medications, and treatment could lead to a longer life for their children as a benefit. Caregivers expressed the perception that the benefits of treatment were enhanced when accompanied with prayer and their faith. For many caregivers, self-efficacy manifest as providing medications to their child: it was the only thing some parents felt they could do for their child.

Relationships developed between healthcare providers and caregivers seemed to influence the understanding of SCD and perceived benefits of medications and treatment at the clinic. Study participants recalled beneficial interactions reflecting a patient-centered care approach by healthcare workers (Table 2). A personal relationship with the healthcare provider was viewed as beneficial in obtaining prioritized treatment and as having an understanding of the child’s clinical diagnosis. Some caregivers expressed mixed feelings and confidence in the health system that was entwined with their economic status. Caregivers with more financial resources sought treatment outside of Liberia or at private health facilities in the hope that they would benefit from more definitive treatment outcomes. Those with fewer financial resources had lower expectations and less value placed of treatment provided by the facility.

## Discussion

This study identified factors influencing the decision-making of caregivers of children with SCD in Liberia. Caregivers identified five key themes that influenced how they perceived and interacted with the clinic following diagnosis. The five themes crossed multiple domains of the socioecological model, highlighting their interaction with multiple levels including family, community, social and cultural norms, and organizational structures. The key determinants of retention in care were grief, stigma, and the existence of support networks. These results include modifiable factors and thus provide a framework for improving retention in care in the future. The quantitative findings of this study reinforced our qualitative findings. Demographic information pertaining to income and poverty levels were reflective of the socio-economic stress surrounding the care of a child with chronic disease. The calculation and use of the IWI provides context for caregiver concerns and the need for low-cost, sustainable interventions. Targeting low-cost modifiable factors such as improving SCD awareness and health communication between healthcare providers and caregivers could impact patient retention.

Lack of education and cultural misconceptions about SCD are commonalties shared across the region [15–17]. Caregivers of children with SCD should understand benefits of preventive care and its contribution to increased lifespan. Increased advocacy and health literacy about SCD are necessary for caregivers to maintain hope. Even in high-income countries, health literacy materials often exceed the recommended reading levels of communities [18]. One study of newborn screening identified as a social barrier the lack of a word for SCD in a local language [19]. Investments in grassroots community advocacy and literacy around SCD and similar chronic diseases could help dispel misconceptions, thereby increasing caregiver engagement and trust with the health system.

Numerous research studies have highlighted the important role hope plays in health outcomes for children and families with severe or chronic illness. Pediatric chronic diseases such as SCD, have physical, psychological, behavioral, and socioeconomic effects that have lasting impact on caregivers [15, 20, 21]. Parents and caregivers of a child with a chronic disease such as SCD may experience life-long sorrow and episodes of sadness [22, 23]. The benefits of integrating mental health services into specialized services for non-communicable diseases has been demonstrated in HIV/AIDS care [24]. Appreciation of the emotional state of the caregiver is crucial to allow providers to deliver patient-centered care. Training institutions and the health system should enable to healthcare providers to support families through grief. In this study, strong faith communities and sense of community provided a source of solace and strength to many caregivers, providing a form of psychotherapy otherwise unavailable in SSA [18, 20, 25]. Mothers are often disproportionately blamed for the disease, enhancing the negative psychological and economic impact of the diagnosis [26]. In settings where patriarchal practices are entrenched, women often bear the burden of taking care of a child with chronic disease. Understanding of the gender dynamic and gender roles in such environments is also critical factor.

Caregivers’ decisions to pursue care are often modulated by the confidence in and relationship between caregiver and provider. In a study of social networks among parents of children with SCD, the most frequently cited sources of support included trusted physicians and other parents [27]. Patient-centered care improves continuity of care and enhances positive perceptions about the program and serves to influence patients’ decision to continue to seek care.

Limited access to care is not readily modifiable, but it is a major systemic factor influencing decision-making and retention in care in Liberia. In this setting, healthcare institutions may be under-utilization because many health needs are met outside of the healthcare system. For example, medications including penicillin and antimalarials, can be purchased from local pharmacies without a prescription. Vaccinations can be obtained in the community through the National Vaccine Program without a clinic visit. Additionally, access to hospital care is limited outside of Monrovia and even on the outskirts of the city. Nearly 40% of infants in Liberia are born outside of a health facility [28]. Whereas primary preventive care is not available to most children in Liberia, follow-up care in the SCD clinic is one of a few opportunities for preventive care. However, the concept may have been unfamiliar to many families in our study. In low low-resource settings, it is imperative to use existing structures and cost-efficient measures to reduce the burden that chronic diseases place on caregivers and families. Decentralizing preventive care is one way of increasing access to vital health services while reducing the burden experienced by parents and caregivers [29].

This study has important limitations. Our sample size was restricted based upon the pilot program that provided participants. Caution should be used when extrapolating findings to other low-resource settings as this study was conducted in one center in Monrovia, Liberia. Second, given the time between diagnosis of child and the time of the study, recall bias may have occurred. Finally, given the gendered dimension of caregiving, future research should consider recruiting equal numbers of caregivers from both genders. Despite these limitations, the qualitative design of the study invites a nuanced discussion of the multifactorial nature of healthcare decision-making from a caregiver perspective. The findings from this study can be used to inform policy and low cost-interventions in similar settings.

Healthcare decision-making is multifactorial and complex however, in a low-resource country such as Liberia there is much that can be done by leveraging existing resources and cultural practices. Initiatives to raise awareness and educate the general public about SCD can help reduce stigma. The health system should decentralize specialty disease knowledge by facilitating training for community-based providers to be familiar with the disease and provide basic services closest to the patient. Increased collaborations between the health system and community and faith leaders to reduce stigma and provide support including psychotherapy to families is a low-cost model that should be further explored. These findings highlight importance of designing sustainable, low-cost interventions that increase caregiver awareness of the benefits of care for children with sickle cell disease and improved healthcare worker communication.

## Supporting information

S1 Fig Consort diagram of recruitment strategy. (TIF)

S1 Text PLOS inclusivity in global research questionnaire. (DOCX)
