# Supplementary figures and images for: Determinants of retention in care of newborns diagnosed with sickle cell disease in Liberia: Results from a mixed-methods study of caregivers
Source: PLOS Glob Public Health. 2023 Apr 4;3(4):e0001705. doi: 10.1371/journal.pgph.0001705 (PMC10072487; doi:10.1371/journal.pgph.0001705)

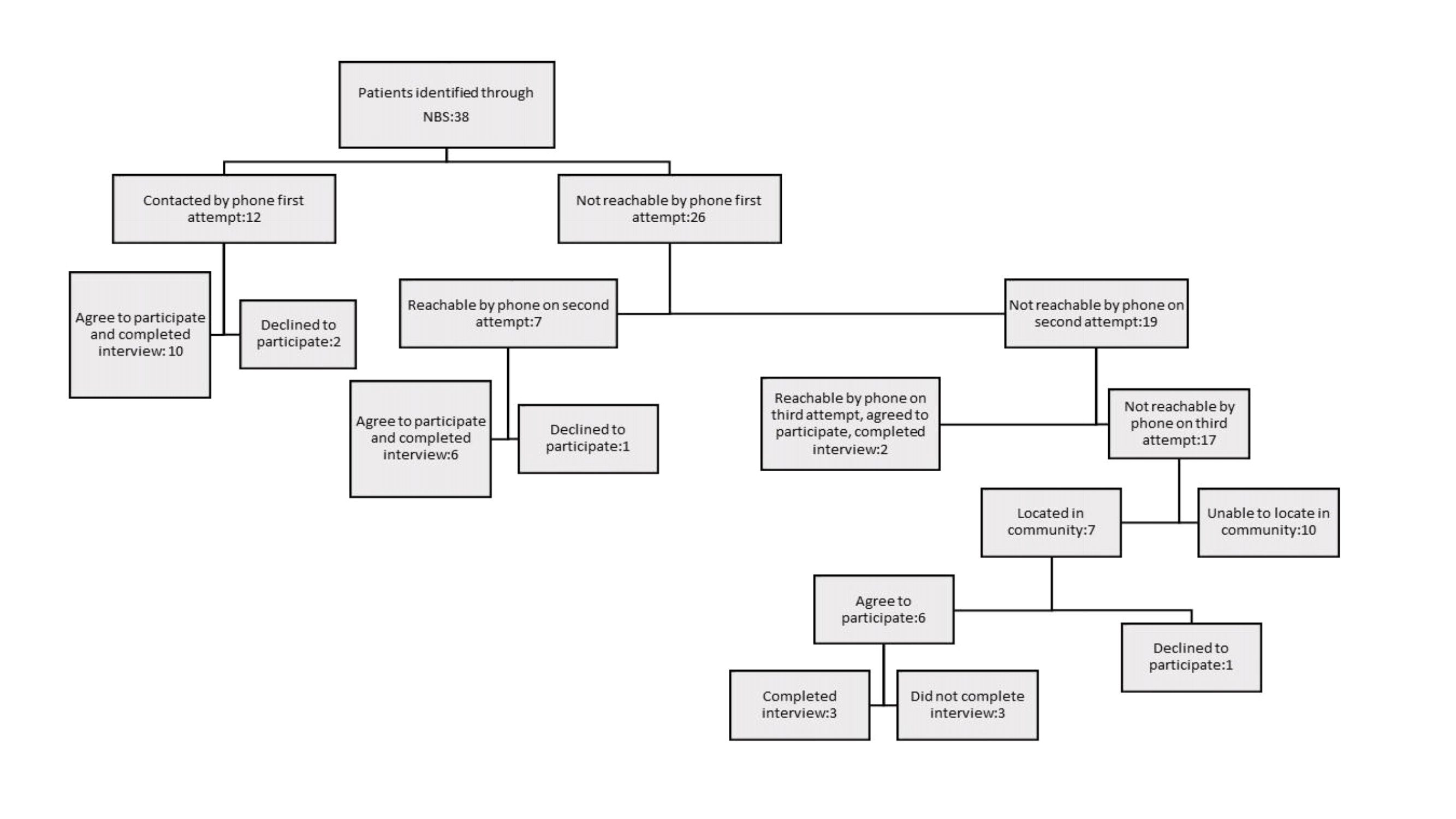

Supplement: S1 Fig — (TIF) [file pgph.0001705.s001.tif]
